# Leprosy presenting as remitting seronegative symmetrical synovitis with pitting oedema syndrome – a case report

**DOI:** 10.1186/s12879-019-4098-9

**Published:** 2019-05-22

**Authors:** Miguel Gomes Guerra, Taciana Marta Ferreira Cardoso Videira, Hugo Alexandre Gomes Morais, Telma Cristiana Resse Nunes Santos, Ricardo Jorge Ferreira Taipa, Miguel Araújo Abreu, Romana Carisa Carvalho Vieira, Diogo Miranda Gonçalves Guimarães da Fonseca, Joana Patrícia Abelha Aleixo dos Santos, Sandra Patrícia Abreu Monteiro Pinto

**Affiliations:** 1 Department of Rheumatology, Centro Hospitalar Vila Nova de Gaia/Espinho, Rua Conceição Fernandes, 4434-502 Vila Nova de Gaia, Portugal; 2 Department of Neurology, Centro Hospitalar Vila Nova de Gaia/Espinho, Rua Conceição Fernandes, 4434-502 Vila Nova de Gaia, Portugal; 3 Neuropathology Unit, Department of Neuroscience, Centro Hospitalar Universitário do Porto, 4099-001 Porto, Portugal; 4 Department of Infectious Diseases, Centro Hospitalar Universitário do Porto, 4099-001 Porto, Portugal

**Keywords:** Leprosy, Remitting seronegative symmetrical synovitis with pitting Oedema syndrome, Peripheral neuropathy

## Abstract

**Background:**

Leprosy typically manifests with skin and peripheral nerve involvement. Musculoskeletal complaints are the third most common, and can be the sole presenting manifestation. They range from arthralgia/arthritis in reactional states to full mimics of systemic rheumatic diseases. Remitting Seronegative Symmetrical Synovitis with Pitting Oedema syndrome has only been described once in a patient with already diagnosed Leprosy.

**Case report:**

A 68-year-old male, from an endemic region of familial amyloid polyneuropathy, presented with an inaugural Remitting Seronegative Symmetrical Synovitis with Pitting Oedema like syndrome, more that 20 years after travelling to Leprosy endemic areas. Arthritis would resurface whenever oral prednisone was tapered, so methotrexate was started, controlling the complaints. Only one year later, after the appearance of peripheral neuropathy and skin lesions, it was possible to diagnose Leprosy, through the identification of *Mycobacterium leprae* bacilli in a peripheral nerve biopsy.

**Conclusion:**

This report is an example of the heterogeneity of manifestations of Leprosy, namely rheumatic, and the challenge of diagnosing it when typical complaints are absent. It is also a reminder that this disease should be considered whenever a patient with a combination of skin/neurologic/rheumatic complaints has travelled to endemic countries in the past.

## Background

Leprosy, or Hansen’s Disease, is an infectious disease caused by *Mycobacterium leprae* (*M. leprae*) that typically affects the skin and peripheral nervous system (PNS) [1]. Known to afflict Humanity since 600 years BC, it is a non-cultivable, obligate intracellular, weakly acid fast pathogen [1] [2]. Ziehl-Neelsen stains may be negative, and Fite Faraco is the best method to identify it; it is also the only bacterial pathogen capable of infecting peripheral nerves [1]. Incubation period is long, ranging from 2 to more than 20 years, with an average of 5 years [3]. Transmission, still not fully understood, appears to occur by skin-to-skin contact or nasal secretions/aerosols [4].

The Ridley-Jopling classification divides Leprosy in 5 categories according to the immunological response and number of bacilli in skin lesions, with a spectrum ranging from tuberculoid to lepromatous [5]. As for the World Health Organization (WHO), patients are classified considering the number of skin lesions and presence of bacilli in skin smear, into paucibacillary (1 to 5 skin lesions, bacteriological index below 2 at all sites) or multibacillary (more than 5 skin lesions; bacteriological index of at least 2 at 1 ore more sites) [6].

Nerve involvement usually occurs early in the disease course, most often with loss of sensory perception, but can also affect the motor nervous system [7]. Considering cutaneous manifestations, these range from flat, sharply defined macules in tuberculoid lesions to diffuse infiltration and indurated plaques and nodules in lepromatous lesions [1]. Despite often belittled, rheumatic complaints can arise, and vary from feeble arthralgia/ to pictures fully mimicking systemic rheumatic diseases [8]. Prevalence is inconstant between studies, ranging between 1 and 78% [2, 9, 10]. Pathogenesis is still not fully understood - proposed mechanisms include reactional states (Types I and II reaction) and direct infiltration of the synovium [2].

The authors report the case of a patient followed at a Rheumatology outpatient clinic for a supposed Remitting Seronegative Symmetrical Synovitis with Pitting Oedema (RS3PE) syndrome that, only after developing polyneuropathy and skin lesions, was diagnosed with Leprosy.

## Case presentation

A 68-year-old previously healthy male presented at a Rheumatology consultation with complaints of hand/feet arthralgia and oedema evolving for more than 6 weeks. He denied fever and there was no history of recent infection or past similar episodes. He worked as a young adult abroad (Iraq, Mozambique, South Africa, and Venezuela) and was natural of an endemic area in Portugal for familial amyloid polyneuropathy (FAP).

Examination revealed swollen and tender bilateral metacarpophalangeal (MCPJ), proximal interphalangeal (PIPJ), tibiotarsal and metatarsophalangeal joints, with pitting oedema of both hands and feet.

Laboratory evaluation revealed an increase in erythrocyte sedimentation rate (45 mm/h) and C Reactive Protein (2.04 mg/dL), with negative rheumatoid factor and anti-citrullinated peptide antibodies. There were no erosions on hand/feet radiography. Hand ultrasound revealed diffuse tenosynovitis of both extensor/flexor compartments, besides joint effusion with doppler sign of MCF and PIF.

Considering the global picture, the diagnosis of RS3PE syndrome was assumed. Symptoms subsided with prednisone 20 mg per day; however, peripheral arthritis relapsed whenever prednisone was tapered.

At this point, an extended workup was performed to exclude hidden neoplastic cause: trans-rectal prostate ultrasound, cervical ultrasound, serum prostate specific antigen, thoraco-abdomino-pelvic computed tomography scan, colonoscopy and upper endoscopy were all normal. The patient then started methotrexate 20 mg/week, with remission of articular complaints and normalization of blood inflammatory parameters.

He stayed asymptomatic for one year, when he started progressive hypostesia/dysestesia of both hands and feet, objectively with loss of sensitivity in glove and sock pattern. Electromyography showed a predominantly sensitive axonal polyneuropathy. No usual causes of polyneuropathy were identified (diabetic, hypothyroidism, alcohol abuse, human immunodeficiency virus, hepatitis B/C and vitamin B12/folate deficiency). Genetic study was performed, bearing in mind the patient’s background and possibility of FAP, revealing no transthyretin gene mutation.

**Fig. 1:**
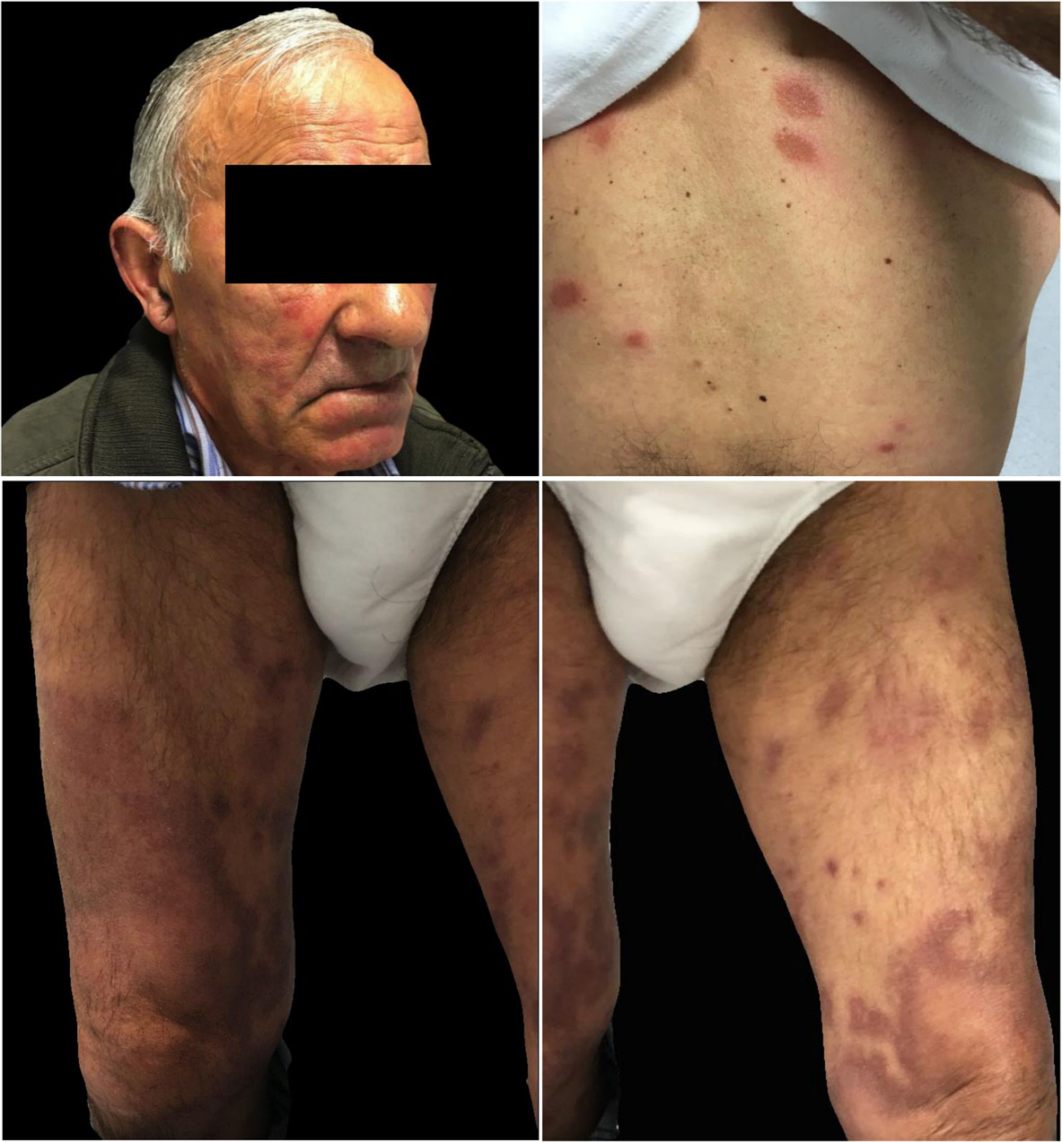
Leprosy skin lesions. Legend **-** Erythemato-violaceous papules and plaques dispersed trough face, dorsum and tights, with large patchy lesions with hyperpigmented margins in both lower limbs

**Fig. 2:**
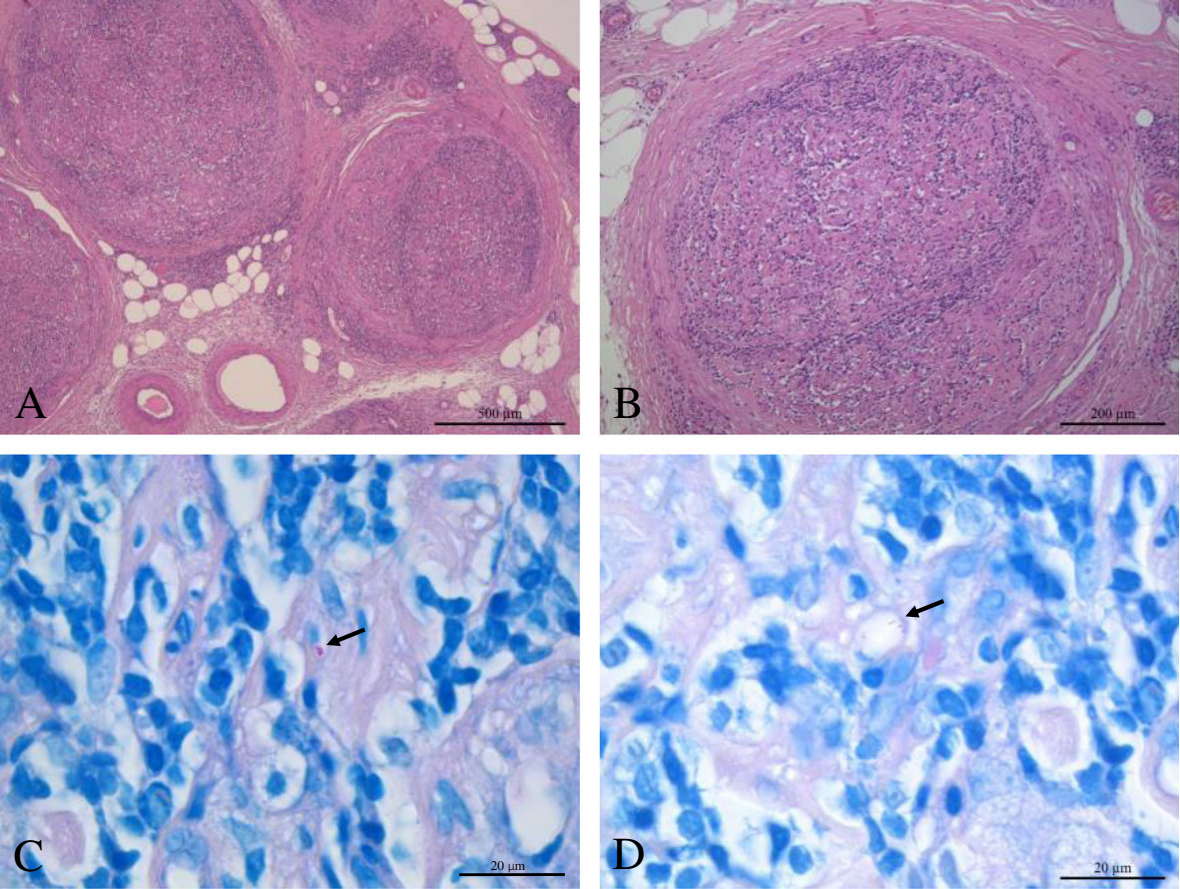
Nerve biopsy histologic study. Legend - Nerve biopsy showing lymphohistiocytic infiltrates, which occupy almost all of the endonerve of the various nervous fascicles observed, and with formation of multiple small non-caseous granulomas (**a** and **b**, hematoxylin and eosin). Fite Faraco staining showed macrophage containing M.leprae bacilli (arrows; **c** and **d**)

Considering the sustained remission of articular complaints, methotrexate was stopped. Neurological symptoms progressively worsened, culminating in the execution of a sural nerve biopsy. Shortly after, erythematous/violaceous hypoaesthetic skin papules and plaques appeared at the trunk, face and limbs, with extensive patch lesions with hyperpigmented margins on both legs (Fig. 1). Skin lesions’ biopsy showed numerous epithelioid granulomas without necrosis, negative for *Mycobacterium tuberculosis* complex DNA. Nerve biopsy, in addition to granulomas, also showed enlarged nerves, with lymphohistiocytic infiltrates and *M. leprae bacilli* in macrophagic vacuoles through Fite Faraco stain (Fig. 2), allowing the definitive diagnosis of multibacillary Leprosy [6].

The patient started treatment with rifampicin 300 mg/month, clofazamin 300 mg/month, dapsone 100 mg/day and clofazamin 50 mg/day. One year after, despite a complete skin lesions regression, there was no improvement in the peripheral neuropathy manifestations.

## Discussion and conclusions

Leprosy is considered a worldwide public health problem, but non-endemic in the patient’s home country (Portugal). According to the latest WHO reports [11], from 2014 to 2015, the prevalence decreased from 0.32 to 0.29 per 10.000 habitants, with a new case detection rate of 3.2 per 100.000. Fourteen countries (from Asia, Africa and South America) declared 95% of the new cases, with India, Brazil and Indonesia in the top 3; 768 foreign-born cases were registered, highlighting the need to a thouroughful evaluation considering non endemic diseases in a world where migration is on the rise.

Rheumatic manifestations of Leprosy are protean and not fully understood. The classical musculoskeletal picture is neuropathic joint, typically in the foot and knee [2, 12], secondary to PNS involvement. Acute, peripheral, remitting polyarthritis is reported in reactional states, but chronic arthritis resembling rheumatoid arthritis can also develop [8]. Sacroiliitis has also been described [13]. Leprosy can even mimic systemic vasculitis, for example, in the context of Lucio phenomenon or cryoglobulinemia [12], or even Systemic Lupus Erythematosus [14] and Systemic Sclerosis [15].

The case presented posed a complex diagnostic challenge. First, a presenting picture of RS3PE syndrome without skin/neurologic complaints is rarely reported in Leprosy. In fact, only one other case labelled as RS3PE syndrome was found in literature, but in a patient already diagnosed with Leprosy [16]. Still, previous case series report a rheumatic manifestation that could also be interpreted as RS3PE syndrome – “swollen hand syndrome”. Paira et al. [17], refer to 10 patients with this term. Prasad et al. [18], in 44 patients, describe 2 with arthritis and swollen hand/feet and 1 with additional tenosynovitis, however without anatomic discrimination of the arthritis and tenosynovitis. Our case followed the typical picture of RS3PE first described by McCarty et al. [19], with pitting oedema and ultrasound reported tenosynovitis/arthritis of both hands and feet in an elderly patient.

Also, despite past history of travels to endemic regions, the incubation period in this case was very long, more than 20 years, making virtually impossible for the clinician that first evaluated the patient, without skin/PNS signs, to suspect of this neglected disease. The favourable response to corticosteroids and immunosuppression was also compatible with the diagnosis of a rheumatic disease. Starting methotrexate may have allowed disease progression, with motor-sensory neuropathy.

Here, another two factors confounded the physicians and delayed diagnosis. First, the patient was from a FAP endemic region. Second, leprosy typically courses with a mononeuritic or multiple mononeuritic pattern. Polyneuropathy is rarer, seen in only 10.5% of the patients with neurologic involvement [20].

Skin lesions appeared in the context of a reverse reaction, early after methotrexate suspension. This could have been the hallmark for diagnosis, as they had typical characteristics. However, once again, the fact that Leprosy is virtually inexistent in the home country delayed correct identification, as Fite Faraco staining and baciloscopy were not performed in the skin biopsy ab initio. Fortunately, nerve histopathology results, available shortly after, confirmed the diagnosis.

In sum, although rheumatic manifestations are common in Leprosy, diagnosing it can pose a puzzling task when they are the sole presenting symptoms, without skin/PNS involvement. It becomes even more challenging in non-endemic countries, with misdiagnoses and delayed treatment. The case presented is unique, combining a presenting picture of RS3PE syndrome with a subsequent chronic polyarthritis, and a place of birth with high incidence of FAP.

## Abbreviations

FAP: Familial amyloid polyneuropathy
*M. leprae*: *Mycobacterium leprae*
MCFJ: Metacarpophalangeal joints
PIP: Proximal interphalangeal joints
PNS: Peripheral nervous system
RS3PE: Remitting seronegative symmetrical synovitis with pitting oedema
WHO: World Health Organization
